# Stereochemical aspects of the nucleophilic attack in different classes of l-asparaginases

**DOI:** 10.1107/S2052252525010826

**Published:** 2026-01-16

**Authors:** Miroslaw Gilski, Kinga Pokrywka, Mariusz Jaskolski

**Affiliations:** a Department of Crystallography, Faculty of Chemistry, Adam Mickiewicz University, Poznan, Poland; b Institute of Bioorganic Chemistry Polish Academy of Sciences Poznan Poland; University of Virginia, USA

**Keywords:** hydro­lases, amido­hydro­lases, reaction mechanisms, structural correlations, stereochemistry, l-asparaginases, Bürgi–Dunitz angle, Flippin–Lodge angle, Herschlag dihedral

## Abstract

l-Asparaginases hydrolyze l-asparagine to l-aspartate with the release of ammonia. Currently, three completely unrelated structural classes of l-asparaginases are known, further subdivided into five types. In each class, the hydrolysis reaction is thought to proceed via a nucleophilic attack of an activated Thr or Ser residue on the carbonyl C*sp*^2^ atom of the substrate amide group. With the possible exception of class 2 l-asparaginases, which function as N-terminal nucleophile (Ntn) hydro­lases, the identity of the nucleophilic residue is, or at least has been historically, the subject of some controversy, and even in class 2 this issue may not be so entirely obvious. Structural chemistry has, however, excellent tools to figure out reaction mechanisms, based on the application of Bürgi’s structure correlation method (SCM). Its principle allows one to predict the reaction trajectory if sufficient structural (crystallographic) examples of the reagents along the reaction path are known. With respect to the nucleophilic attack on a carbonyl group, the stereochemistry is governed by the nucleophile⋯electrophile distance and the Bürgi–Dunitz angle, later supplemented with the Flippin–Lodge angle. The latter angle is shown to be a poor parameter and is better replaced by the Herschlag dihedral between the planes of the attacking nucleophile and the attacked electrophile. In structural enzymology, applicability of the SCM principle requires the availability of structural examples of the enzyme in question in complex with the substrate or product of the catalytic reaction. In this work, we applied the SCM concept to the three classes of l-asparaginases, identifying in each case the most probable nucleophilic residue as Thr12 in EcAII (class 1), Thr179 in EcAIII (class 2) and Ser48 in ReAV (class 3). In addition, we applied the SCM analysis to the newly identified group of asparaginases without proper classification, called short-chain asparaginases, providing a basis for their proper affiliation in class 1. Finally, the SCM analysis shows that the chirality of the nucleophilic attack in class 2 asparaginases (pro-*R*) is opposite to that in all other asparaginases.

## Abstract 2

Stereochemical analysis based on the principle of structure correlations has been carried out to identify the primary nucleophile residue in each of the three classes of l-asparaginases and in a group of classless enzymes, helping to confirm their classification.

## 1. Introduction

**Figure 1:**
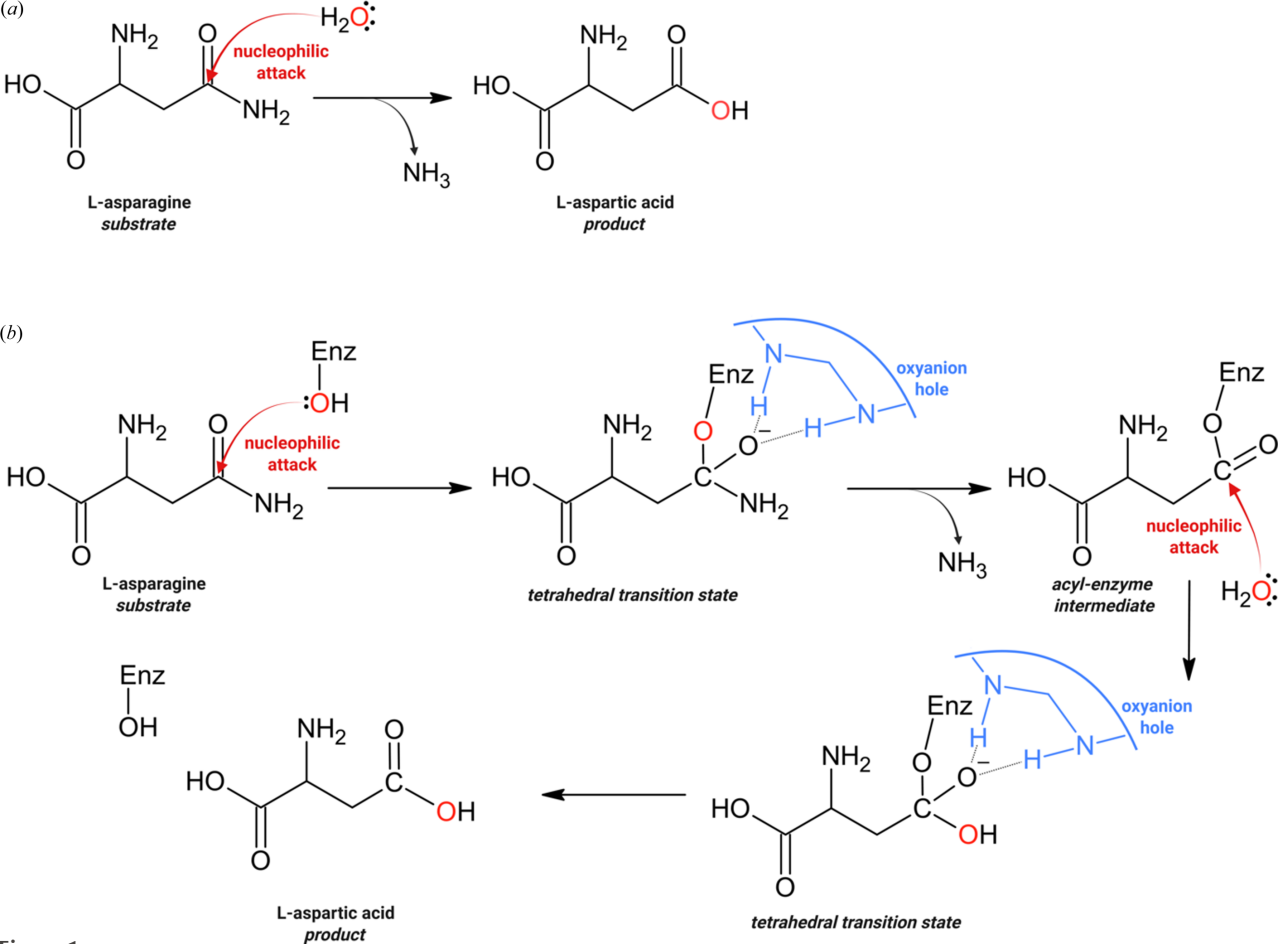
Schematic representation of the (*a*) single-displacement or one-step and (*b*) double-displacement or two-step l-asparaginase reaction. The nucleophilic centers are in red. ‘Enz’ stands for ‘enzyme’.

The principle of structural correlations, also known as the structure correlation method (SCM), was conceived by Hans-Beat Bürgi (1973 ▸), who noted that if reacting moieties are found together in many crystal structures, then the variable ‘crystal field’ that they experience can be assumed to force them on a path that maps the stereochemistry of their reaction coordinate and leads along a potential-energy trough in the potential-energy landscape. The SCM principle was later greatly developed and applied to numerous chemical transformations by such authors as Jack D. Dunitz (Dunitz & Bürgi, 2008 ▸) or Gastone Gilli (Ferretti *et al.*, 1994 ▸). One of the first notable applications of the SCM principle was to the nucleophilic addition to a carbonyl group of a trigonal moiety with an electrophilic C*sp*^2^ center, which in structural enzymology may be equivalent to the nucleophilic attack on a carbonyl-containing substrate. Such a nucleophilic attack by an activated nucleophilic group of the enzyme is thought to be the first step of many hydrolytic reactions that break the amide bond of a peptide chain (proteases) or at the side chain of l-Asn (asparaginases) or l-Gln (glutaminases). The nucleophile is usually a serine, threonine or cysteine residue, and the activation is provided by a strong Lewis base, efficiently attracting the proton from the O/S—H nucleophile. In this (prevalent) type of mechanism (also known as the double-displacement mechanism), the nucleophilic attack leads through a tetrahedral transition state to a β-acyl-enzyme (ester) intermediate, which is ultimately hydrolyzed by an activated water molecule in the second step of the reaction. Another possibility, not covered in this paper but used, for example, by aspartic proteases, is direct activation of a water molecule, which then completes the hydrolysis reaction in one step (Fig. 1 ▸).

**Figure 2:**
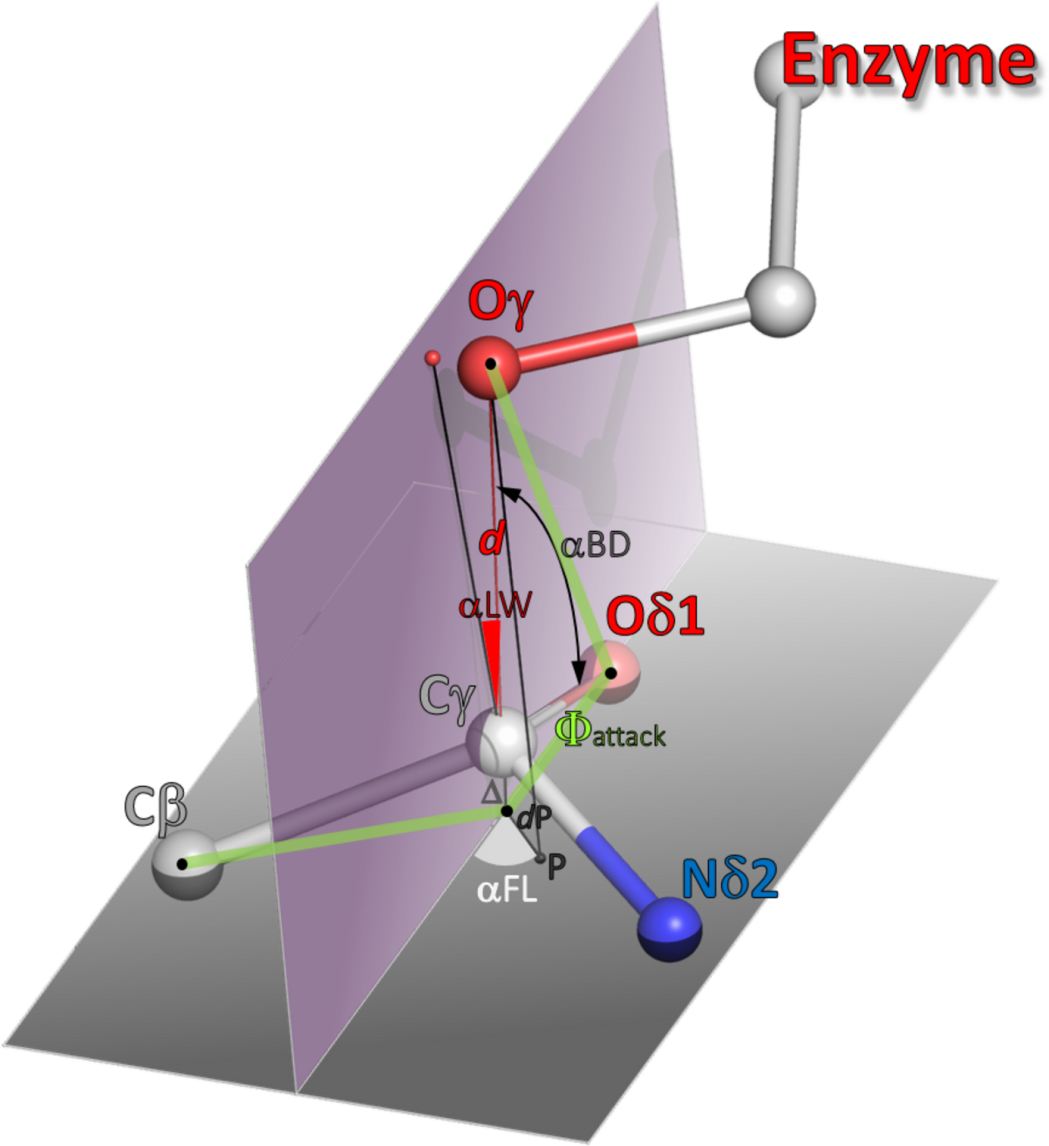
Definition of the geometrical parameters describing the stereochemistry of the attack of an enzyme hydroxyl nucleophile on the amide group electrophile of l-asparagine. The atoms are labeled as in a real l-asparaginase case. For some of the general definitions, Oγ would correspond to O_nuc_ and Cγ to C_el_. When the pyramidalization Δ of the amide group is significant, the projection of the Cγ atom on the plane of its O/N/C substituents is marked as C′ (black point below Cγ). α_BD_ is the Bürgi–Dunitz angle O_nuc_–C_el_=O; α_FL_ is the Flippin–Lodge angle defined as 180° − P–C′=O, where P is a projection of O_nuc_ on the electrophile plane; α_LW_ is the Lubkowski–Wlodawer angle O_nuc_–C_el_–P′, where P′ is a projection of O_nuc_ on plane Ω, which is perpendicular to the electrophile plane through the C=O bond; Φ_attack_ is the O_nuc_–O=C′—C torsion angle; *d* is the O_nuc_⋯C_el_ distance; *d*P is the P⋯C′ distance; the degree of pyramidalization Δ is defined as the deviation of C_el_ from the plane of its O/N/C substituents, *i.e.* C′⋯C_el_; Δ > 0 when C_el_ deviates toward O_nuc_.

In their analysis of the O_nuc_⋯C_el_=O interactions (nuc/el stand for nucleophile/electrophile) in crystals, Bürgi, Dunitz & Shefter (1974 ▸) (hereafter denoted as BDS) established that the α_BD_ angle of approach of the nucleophile to the C=O bond, expressed as O_nuc_⋯C_el_=O, should be 105 ± 5°. Later, this value was refined as ∼90° (Rzepa, 2015 ▸; Radisky & Koshland, 2002 ▸). Heathcock & Flippin (1983 ▸) realized that the α_BD_ angle alone is insufficient to fix the nucleophile in space relative to the electrophile plane and introduced the Flippin–Lodge angle, α_FL_, defined by projecting the nucleophile atom onto the electrophile plane (point P) and measuring the angle (azimuth) of the C_el_–P line from the extension of the O=C_el_ bond. The expected value of the α_FL_ angle is ∼0 ± 7° (Fig. 2 ▸). In a recent paper, Du *et al.* (2025 ▸) presented a very comprehensive analysis of the reaction mechanism of serine proteases, which also use a Ser nucleophile to hydrolyze the peptide bond, in which the nucleophilic attack is described by the α_BD_ angle (denoted as α_attack_) and a dihedral angle Φ_attack_ between the O_nuc_⋯O=C_el_ plane and the plane of the electrophile, defined as the plane of the three substituents (=O, —N, —C) at the electrophilic C_el_ center. This proposition somehow overlooks and replaces the earlier Flippin–Lodge definition, but in fact appears to be more handy (*e.g.* it avoids the indeterminacy of α_FL_ when the O_nuc_ atom is directly above the C_el_ center), while still defining the reaction geometry adequately. The optimal value of Φ_attack_, obtained from both the experimental observations and theoretical calculations, given by Du *et al.* (2025 ▸) is 84°.^1^ In this work, we will characterize the nucleophilic addition to the amide group of l-asparagine using both parametrizations. However, instead of using the complicated (and rather artificial) torsion angle ‘normalization’ (to within 0–90°) proposed by Du *et al.* (2025 ▸), we will calculate Φ_attack_ as a standard O_nuc_—O=C′—C torsion angle, where C′ is the projection of the C_el_ atom on the electrophile plane, defined by its O/N/C substituents.

The subject of this paper is the asparaginase reaction, which converts l-Asn to l-Asp and ammonia. There are three completely unrelated structural classes of l-asparaginases, further subdivided into types I–V (Loch & Jaskolski, 2021 ▸). Class 1 asparaginases (formerly bacterial asparaginases) are homotetrameric enzymes of two types (I and II) that are structurally very similar but quite different enzymatically. Their enzymatic efficiency is so different that the type II enzymes, *e.g.* EcAII, with micromolar substrate affinity, are potent antileukemic drugs, while the type I enzymes, *e.g.* EcAI, with millimolar affinity, are not. EcAI and EcAII are both *Escherichia coli* enzymes. Their active sites contain a T-K-D triad similar to the S-H-D triad of serine proteases, which would seem to automatically indicate the Thr nucleophile. However, opposite the T-K-D triad (relative to the substrate molecule) and located on a flexible gating element (FGE), there is another Thr residue, which in the closed FGE conformation is even better poised as the primary nucleophile. Class 2 asparaginases (formerly plant or type III asparaginases) are homodimeric Ntn-hydro­lases that become active after autoproteolytic cleavage, which liberates the nucleophilic Thr residue at the N terminus of the new β subunit (Brannigan *et al.*, 1995 ▸). While the nucleophilic Thr residue of the Ntn-hydro­lases would appear to be quite obvious, the active site of class 2 asparaginases also contains other Thr residues, whose potential involvement in the catalytic mechanism should be at least tested. Class 2 asparaginases have millimolar l-Asn affinity. An interesting feature of class 2 asparaginases is that the activity of some of them is boosted by potassium cations (Bejger *et al.*, 2014 ▸). Class 3 asparaginases, originally identified in the symbiotic *Rhizobium etli* bacterium capable of atmospheric N_2_ fixation, are the newest addition to the family. They are subdivided into two types (IV and V) of very similar structure and active site (Loch *et al.*, 2021 ▸, 2023 ▸). The active site is comprised of two S-K tandems and a nearby Zn^2+^ cation coordinated by one Lys, two Cys residues and a water molecule. The metal cation does not take direct part in the catalytic reaction but anchors the l-Asn substrate through coordination of its α-amino and α-carb­oxy groups (Pokrywka *et al.*, 2025 ▸). Class 3 asparaginases have substrate affinity in the low millimolar range. There are only three crystal structures in the Protein Data Bank (PDB; Berman *et al.*, 2000 ▸) with a literature reference (Pokrywka *et al.*, 2025 ▸) of a class 3 asparaginase (ReAV) in complex with its l-Asn substrate, corresponding to two site-directed mutants, one of which is an apo form lacking the metal cofactor, and to the wild-type enzyme. To enhance this meager set, we included in this work an unpublished high-resolution reference crystal structure of a cadmium-containing ReAV K138A mutant in complex with l-Asn (PDB ID 9sdk).^2^ The cadmium cation, as a replacement for the native zinc cation, slows the reaction down, allowing capturing of the enzyme–substrate complex in the crystal.

The goal of this work is to use Bürgi–Dunitz analysis to provide stereochemical confirmation of the identity of the catalytic nucleophile in all three classes of asparaginases. With regard to class 1, such an analysis has already been attempted by Lubkowski & Wlodawer (2019 ▸), who concluded that the primary nucleophile is the Thr residue opposite the T-K-D triad. Unfortunately, in their work the authors used an incorrect definition of the α_FL_ angle, by projecting the nucleophile-approach line onto a plane perpendicular to the electrophile system passing through the C=O bond, and measuring the angle between the nucleophile-approach line and this projection. We will call this angle the α_LW_ angle. It becomes quite useful when the true α_FL_ angle cannot be calculated because of (nearly) exact orthogonality of the nucleophilic attack. When the O_nuc_⋯C_el_ line of attack is indeed perpendicular to the electrophile plane, then the expected value of α_LW_ is, of course, 0°. To make the treatment of class 1 mathematically correct and consistent with the α_BD_/α_FL_/Φ_attack_ parametrization, and to subdivide it into type I and type II, we will include this class (expanded to include the most recent PDB cases) in our analysis as well.

The stereochemistry, including chirality, of the assumed nucleophilic attack is well defined if the attacked ligand is the l-Asn substrate. The situation would appear to be ambiguous with the l-Asp (product) ligand, which has two O atoms at the side-chain functional group. However, the ambiguity can be resolved easily because of the oxyanion hole, which is a characteristic feature of amido­hydro­lases, usually consisting of two N—H or similar hydrogen-bond donor groups, and which functions to stabilize the negative charge that develops on the carbonyl O atom of the substrate in the tetrahedral transition state (Henderson, 1970 ▸; Robertus *et al.*, 1972 ▸; reviewed in Wlodawer *et al.*, 2024 ▸) of the reaction. In other words, the oxyanion hole, well known in each l-asparaginase class, identifies the carbonyl Oδ1 atom of the substrate, unequivocally pointing to where the substrate NH_2_ group (Nδ2 atom) should be. Having such a unique pose of the substrate amide, it is possible to establish the *R*/*S* chirality of the tetrahedral species in the transition state. From the cases analyzed by Lubkowski & Wlodawer (2019 ▸), this chirality in class 1 is *S*.^3^ In other words, if we take a bird’s eye view of the electrophile plane from the vantage point of the approaching nucleophile in such a way that the Cβ substituent of the (transiently) tetrahedral center is pointing straight ahead of us, then the NH_2_ substituent is on the left and the ‘carbonyl O atom’ on the right. It will be interesting to check whether this chirality rule also holds in class 2 and 3 l-asparaginases.

For some class 1 and class 2 enzymes there are crystal structures of the covalent β-acyl-enzyme ester intermediates. Examples are provided by the PDB structures 4eca (Palm *et al.*, 1996 ▸) or 6v24/6v25/6v27/6v28 (Lubkowski *et al.*, 2020 ▸) in class 1 and 8c0i (Janicki *et al.*, 2023 ▸) or 4o0h (Nomme *et al.*, 2014 ▸) in class 2. Obviously, when the covalent intermediate is known, it automatically indicates the primary nucleophile. Therefore, in principle, no additional testing of alternative nucleophiles is necessary in such cases. However, one should be aware that when the active site has been genetically manipulated, for example, by mutating one of the candidate nucleophiles, the other nucleophile may rescue the catalytic reaction, thus leading to wrong conclusions. Anyway, the flat ester groups are not suitable for envisioning the tetrahedral nucleophilic attack, and thus have to be omitted from our analysis. On the other hand, there are a few structures of class 1 asparaginases in the PDB where the reaction has been arrested in the tetrahedral transition state. Naturally, we have included these cases, together with the alternative nucleophile candidates, in order to establish the cogency of the Bürgi–Dunitz test by comparing known positive and negative outcomes.

Our analysis also includes the newly discovered short-chain (or ‘short’) classless l-asparaginases, with sequence and fold similarity to class 1 enzymes but devoid of the C-terminal domain (Zhang *et al.*, 2024 ▸), which is largely considered to be the oligomerization domain. Nevertheless, the short proteins also form homotetramers (albeit quite different from the classic class 1 tetramers) and have active sites consistent with class 1 classification. The Tyr residue that distinguishes type I and II active sites in class 1 (Janicki *et al.*, 2025 ▸) comes from an adjacent protomer. We have provisionally classified the short asparaginases in class 1 as type s.

In contrast to small molecules, the application of the SCM method to macromolecules is hampered by the massive use of stereochemical restraints, which may force a geometry that is thought to be ‘ideal’ but is in fact in conflict with the actual stereochemistry assumed by the free reagents. An example of such artificial strain could be the enforcement of strict planarity of the electrophile group, preventing its pyramidalization toward the nucleophile. Likewise, the carbonyl C=O bond is usually tightly restrained, so that its lengthening upon a nucleophilic addition reaction is unlikely to be prominent in macromolecular crystal structures. However, softer parameters, such as bond angles and especially torsion angles, are less susceptible to such ‘distortions by idealization’, and thus angular correlations may stand out if large data sets are used. This is especially true at higher resolution, when the diffraction data clearly dominate over the geometric restraints, to the point that at atomic resolution only disordered fragments may need stereochemical restraining (Jaskolski, 2017 ▸). For this reason, only structures with at least 3 Å resolution were selected for the present analysis.

Finally, we want to point out that our stereochemical analysis of the l-asparaginase reaction is a prelude to an even more comprehensive analysis, where sufficiently highly populated ensembles of the reactants on the reaction path would allow us to figure out the kinetic parameters of the reaction, in an approach analogous to that presented for 1231 structures of serine proteases by Du *et al.* (2025 ▸). At present, such a kinetic extension is not yet possible, because of too few known crystallographic structures in the PDB, especially for class 2 and 3 l-asparaginases.

## 2. Methods

### 2.1. Data mining

The data set of crystal structures of l-asparaginases in complex with either the l-Asn substrate or the l-Asp product of the reaction was created from the PDB inventory presented by Wlodawer *et al.* (2024 ▸), with additional entries covering the period up to 15 January 2025. The final subset of structures was arrived at after the application of additional filters. (i) Only structures with resolution of at least 3 Å were retained. (ii) The uniqueness of the data set was guaranteed by a manual check for the absence of accidental PDB duplicates (Wlodawer *et al.*, 2025 ▸). A decision had to be made as to whether redundant structures of identical complexes crystallized isomorphously in identical conditions (*e.g.*6pa3/6pa8) presented by Lubkowski & Wlodawer (2019 ▸) should be removed or not. Ultimately, we decided, like Lubkowski & Wlodawer (2019 ▸), to keep both structures, arguing that they were determined using independent sets of experimental observations. (iii) Active sites with either the ligand or the nucleophile disordered were excluded; an exception to this rule was two class 1 cases of type II, where an alternative conformation corresponded to a covalent tetrahedral transition state, clearly modeled in the electron density. (iv) Structures with ligands other than asparagine or aspartic acid were excluded, in contrast to Lubkowski & Wlodawer (2019 ▸), who retained ligands such as succinic acid. We did include, however, l-Gln/l-Glu complexes in order to study the possibility of the related l-glutaminase reaction. Complexes with the enantiomeric d-Asn/d-Asp were retained for the purpose of comparing their stereochemistry with that of the proper l-configuration substrate. Lists of the PDB accession codes of the complexes used in this analysis are provided in Supplementary Table S1.

Analysis of each entry began with a careful manual check of each ligand binding site, orientation of the ligand functional group (C_el_ and its substituents), and positions of potential nucleophiles (O_nuc_) and the oxyanion hole in the active site area. If the true ligand was originally l-Asp, one of the Oδ atoms was treated as Nδ2 based on the indication of the Oδ1 atom provided by the oxyanion hole.

Such verified atomic coordinates were then fed to a Python program, called *BD*, that calculated the α_BD_, α_FL_ and α_LW_ angles, as well as some other geometrical parameters, such as the Φ_attack_ torsion angle, C_el_ pyramidalization Δ, O_nuc_⋯Cγ distance *d*, and the length of the C_el_–P vector, *d*P. Pyramidalization of the carbonyl C*sp*^2^ atom of the substrate/product was calculated as the deviation of that atom out of the plane of its three substituents. Deviation in the direction of the assumed nucleophile was defined as positive. The chirality (chiral volume) of the potential tetrahedral transition state was calculated under the assumption that the O_nuc_ atom is the fourth substituent of the C_el_ chirality center, which is equivalent to moving the C_el_ atom to the O_nuc_ position. The calculated chirality was checked manually in *PyMOL* (DeLano, 2002 ▸) and in the *BD* program.

We note here that when the C_el_–P vector is close to zero, *i.e.* the O_nuc_⋯C_el_ line is essentially perpendicular to the electrophile plane, then the estimation of the α_FL_ angle becomes unreliable. When the O_nuc_⋯C_el_ line is exactly perpendicular to the electrophile plane, the α_FL_ angle becomes indeterminate and cannot be calculated.

### 2.2. Confirmation of the correct orientation of the substrate amide group

For the l-Asp complexes, we systematically renumbered (if necessary) the side-chain Oδ1/Oδ2 atoms in such a way that the Oδ1 atom was inserted in the oxyanion hole (well known in each class), *i.e.* was playing the role of the carbonyl O atom of the substrate. We note that Lubkowski & Wlodawer (2019 ▸) analyzed the atomic displacement parameters (ADPs) for this purpose. The method used here is more robust and unequivocal. In 100 cases the Oδ1/Oδ2 atoms of the l-Asp side chain had to be swapped. A similar check was applied to the amide group of the l-Asn substrates, to make sure that the Oδ1 atom was always directed toward the oxyanion hole, but all instances were found to have the correct orientation.

### 2.3. Computer programs

A dedicated code called *BD* has been developed to support the geometrical calculations. *BD* is written in Python using the Google Colaboratory environment. It performs all key calculations for the presented analysis. The program is divided into two main parts, located in two Colab Notebook cells. The first part contains a simple interface for data input (the pathname of the coordinate file in legacy PDB format, the names and residue numbers of the nucleophile and substrate residues) and for handling the output files. The second Notebook cell contains the main computational module and includes interactive visualization of the active sites. The calculations and visualization are performed for all protein chains in the model. During the first run, the program installs and loads all required standard and external modules, including *py3Dmol* (Python wrapper for *3Dmol* JavaScript library, https://pypi.org/project/py3Dmol/; Rego & Koes, 2015 ▸), *scikit-spatial* (spatial objects and computations based on NumPy arrays, https://scikit-spatial.readthedocs.io/en/stable/) and *Biopython* (Cock *et al.*, 2009 ▸). The program displays an interactive 3D model of the structural fragments involved in the nucleophilic attack, which is indicated by a green arrow. Clicking on any atom zooms and centers the view and shows the full atom label. After visual inspection, clicking the *Run* button generates a simple list of the results and saves them to .docx and .csv files. The *BD* program is available from MG on request.

All structural illustrations were prepared in *PyMOL* (DeLano, 2002 ▸) and annotated in *BioRender* or *CorelDRAW* (2004 ▸).

### 2.4. Definition of the parameters used in this work

The geometrical parameters characterizing nucleophilic addition of a hydroxyl group to the amide moiety at the side chain of l-asparagine are illustrated in Fig. 2 ▸ and are briefly described below:

α_BD_, Bürgi–Dunitz angle, O_nuc_–C_el_=O;

α_FL_, Flippin–Lodge angle, 180° − P–C′=O, where P is a projection of O_nuc_ on the electrophile plane and C′ is a projection of C_el_ on the plane of its O/N/C substituents;

α_LW_, Lubkowski–Wlodawer angle, O_nuc_–C_el_–P′, where P′ is a projection of O_nuc_ on a plane (Ω) perpendicular to the electrophile through the C=O bond;

Φ_attack_, O_nuc_–O=C′—C torsion angle;

*d*, O_nuc_⋯C_el_;

*d*P, P⋯C_el_;

Δ, pyramidalization of the electrophile plane, *i.e.* C′⋯C_el_, Δ > 0 is for deviation of C_el_ toward O_nuc_.

To describe the distributions of the geometrical parameters, we will use histograms, as well as parametric descriptions in the form of mean (μ) and standard deviation (σ), assuming normal distributions. The normal distributions are quite evident for class 1, where there are many observations. By analogy, they were also assumed for classes 2 and 3. In our notation the statistical parameters are written as μ (σ), where σ is given as explicit value (*i.e.* differently from typical crystallographic notation).

## 3. Results and discussion

### 3.1. Preliminary statistics

**Figure 3:**
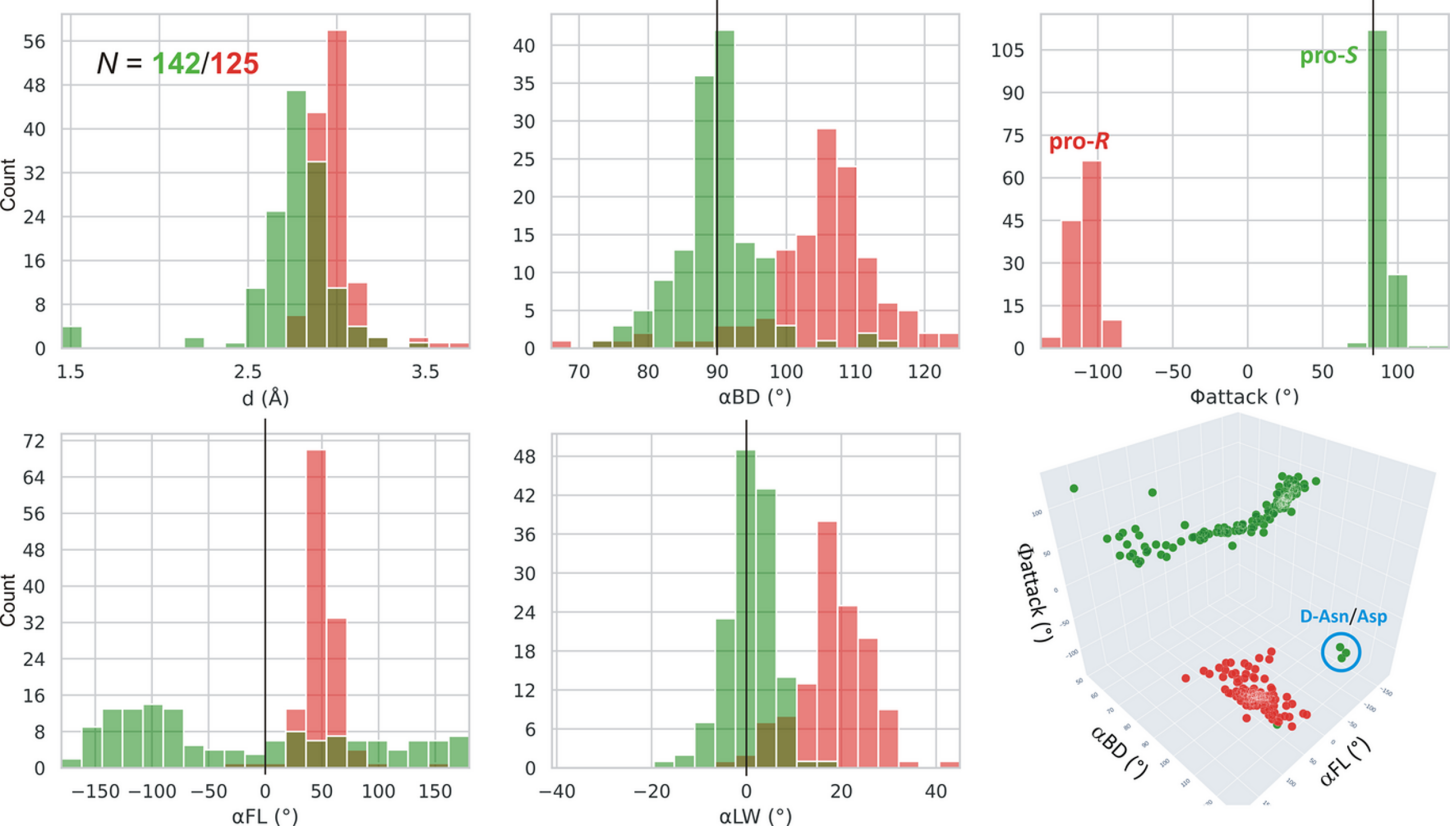
Distribution of parameters *d*, α_BD_, Φ_attack_, α_FL_ and α_LW_ for the more (Thr12, green) and less (Thr89, red) likely nucleophile in class 1 l-asparaginases, including types I and II. Residue numbering of the reference protein EcAII is used. The vertical black lines mark the expected values of α_BD_, α_FL_, α_LW_ and Φ_attack_. The figure shows that the α_FL_ parameter is of little use, as it is widely scattered owing to its poor definition (perpendicular orientation of the O_nuc_⋯C_el_ vector relative to the electrophile plane). The α_LW_ angle is correlated with α_BD_, and will not be used in further analyses. The last panel shows a 3D correlation scatterplot of α_BD_, α_FL_ and Φ_attack_. In addition to well defined clusters for Thr12 (green) and Thr89 (red), there are a few green outliers (lower right corner) corresponding to the wrong d-Asn substrate.

In each l-asparaginase class, there are two Thr/Ser residues to be considered as the potential primary nucleophiles. In class 1 the pair is Thr12/Thr89 (Lubkowski & Wlodawer, 2019 ▸), in class 2 Thr179/Thr230 (Michalska *et al.*, 2005 ▸), and in class 3 Ser48/Ser80 (Loch *et al.*, 2021 ▸), in the numbering of the reference enzymes EcAII, EcAIII, and ReAV, respectively. We created, therefore, simple histograms for each pair and for the most indicative parameters *d*, α_BD_, α_FL_, α_LW_ and Φ_attack_ to see whether these parameters can clearly distinguish between these nucleophile ambiguities. The results for class 1 (including type s) are presented in Figs. 3 ▸ and 4 ▸, and for classes 2 and 3 in Figs. 5 ▸ and 6 ▸, respectively. After a preliminary analysis in class 1 (Fig. 3 ▸), the set of parameters was narrowed to *d*, α_BD_ and Φ_attack_, as explained below.

### 3.2. Class 1 asparaginases revisited

In class 1 asparaginases the potential nucleophiles are two Thr residues – one a part of the T-K-D triad, and one located on the FGE loop, Thr89 and Thr12, respectively, in EcAII numbering (Fig. 7 ▸). Curiously enough, the oxyanion hole of EcAII is formed by the main-chain N—H amide groups of these two potential nucleophiles, Thr12 and Thr89 (Fig. 8 ▸). We will be using these residue numbers with reference to other class 1 proteins as well. For adequate evaluation of the Thr12 residue, it must be properly ordered and defined in the crystal structure (full occupancy, ADPs < 100 Å^2^). The controversy about whether the primary nucleophile of EcAII, and by extension in all class 1 l-asparaginases, is Thr12 or Thr89 was resolved in favor of Thr12 by the crystal structure of a Thr12-acyl enzyme intermediate (Palm *et al.*, 1996 ▸) and by the Bürgi–Dunitz analysis published by Lubkowski & Wlodawer (2019 ▸). We repeat a similar analysis here for the following reasons: (i) the previous authors used an incorrect definition of the Flippin–Lodge angle; (ii) there are new PDB cases available; and (iii) our analysis separates type I and type II enzymes to see whether the differences in the composition of their active sites might be reflected in their reaction stereochemistry.

A summary of the results for class 1 with subdivision into type I and type II asparaginases is presented in Supplementary Table S2. Overall, there were 142 cases of l-Asn/l-Asp complexes in this class, subdivided into 14 of type I and 128 of type II. Distinction between type I and type II asparaginases is usually made based on sequence alignments with the EcAI and EcAII templates (*e.g.* Zielezinski *et al.*, 2022 ▸). However, in view of the relatively contiguous spectrum of sequences between the EcAI and EcAII templates, which makes distinction of border cases difficult, an even better marker is the active-site Tyr residue (Tyr25 in EcAII numbering), which in type II enzymes comes from the same subunit (and even the same FGE element) as the Thr12 nucleophile, while in type I it is contributed by the complementary subunit of the intimate dimer (Janicki *et al.*, 2025 ▸) within the tetrameric assembly. The present analysis includes 14/101 type I/II cases from the Lubkowski & Wlodawer (2019 ▸) analysis and 0/27 additional cases from the Lubkowski & Wlodawer (2021 ▸) survey. Among the class 1 cases (including type s, see Section 3.5) in our data set, there were several cases where the FGE was in open conformation and the Thr12 nucleophile was far away (*d* > 10 Å) from the substrate. We eliminated such cases manually, setting a *d* ≤ 4 Å limit for Thr12. It is not clear whether Lubkowski & Wlodawer (2019 ▸) applied a similar criterion in their analysis.

**Figure 4:**
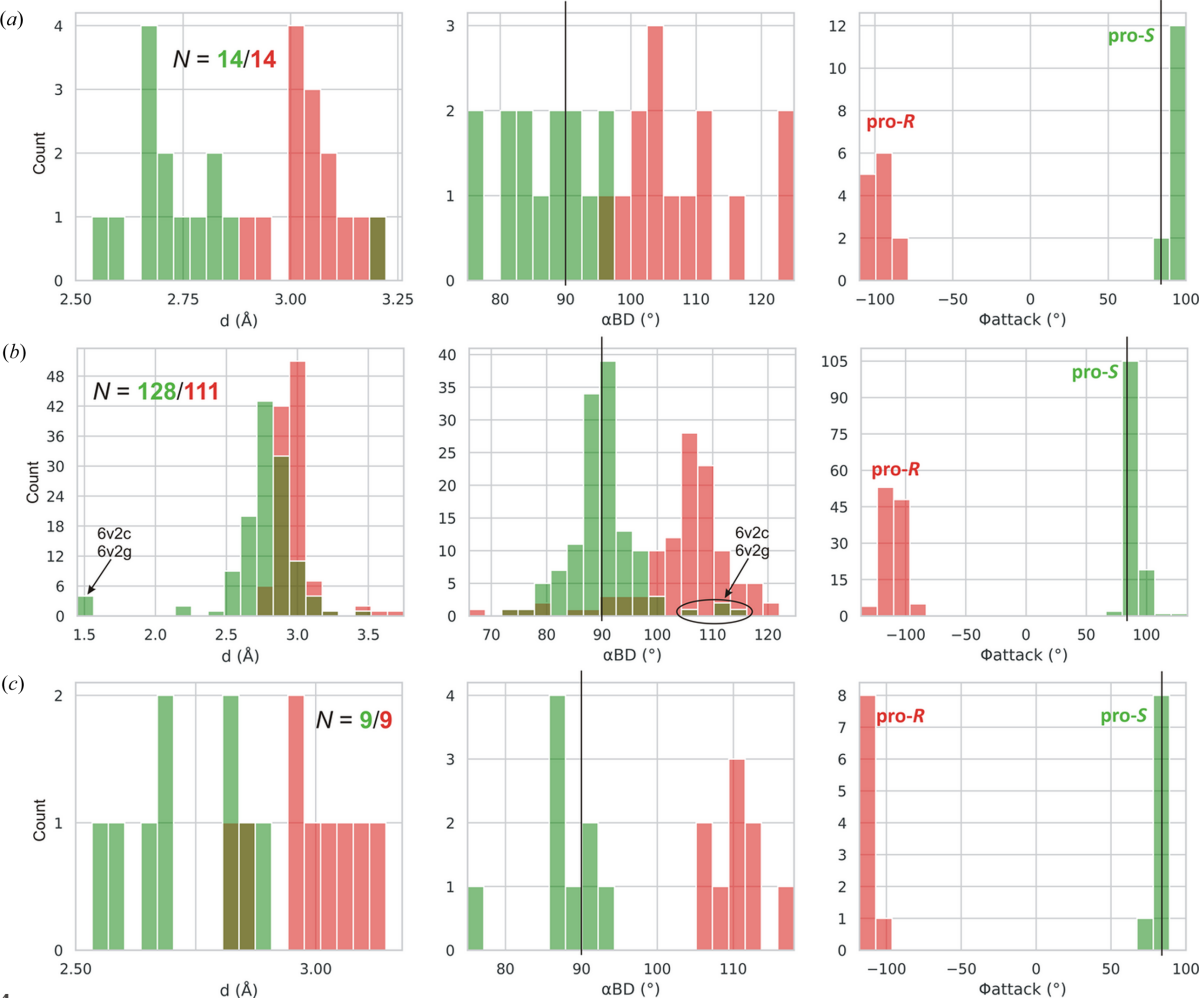
Histograms presenting the distribution of parameters *d*, α_BD_ and Φ_attack_ for the more (Thr12, green) and less (Thr89, red) likely nucleophile in subtypes of class 1 l-asparaginases as follows: (*a*) type I, (*b*) type II, (*c*) type s. Residue numbering of the reference protein EcAII is used. The vertical black lines mark the expected values of α_BD_ and Φ_attack_.

Fig. 3 ▸ presents consolidated histograms combining type I and type II l-asparaginases of class 1 for the main stereochemical parameters considered in this work. It shows that the α_BD_/α_LW_/Φ_attack_ angles unambiguously indicate Thr12 as the nucleophile, with the mean values (and standard deviations, given in parentheses) of 90 (6)/0 (6)/90 (6)°. The corresponding averages in the type I and type II subsets are 87 (7)/−2 (4)/92 (4)° and 90 (6)/1 (6)/89 (6)°, respectively. There is, therefore, no significant difference between the geometry observed for types I and II. The O_nuc_⋯C_el_ distance *d* for Thr12 is systematically shorter than for Thr89. The α_FL_ parameter is practically of no use, as it covers the whole angular range without any distinct peak. The α_LW_ angle shows a sharp distribution but since it is correlated with the α_BD_ angle (see *Conclusions*[Sec sec4]), it will be omitted from our further analysis as well. Analogous histograms for subtypes I and II in class 1 are shown in Fig. 4 ▸ and the mean values of the geometrical parameters are collected in Table 1 ▸. The mean values of the α_BD_/α_LW_/Φ_attack_ parameters agree well with the expected values of 90/0/84°, although the Φ_attack_ values are closer to the average of ∼90° attributed to BDS.

In the type II category there are four instances representing true covalent tetrahedral transition states (*i.e.* the end state of the nucleophilic attack considered in this analysis) at the Thr12 residue, marked on Fig. 4 ▸(*b*) using their PDB ID codes. The *d* parameter is then of course equivalent to a covalent bond (∼1.5 Å) and the Φ_attack_ angle has its expected value. The α_BD_ angle is, however, somewhat modulated, with a value of 110°.

The positive value of the Φ_attack_ angle (*ca* +90°) defines the chirality of the nucleophilic attack in class 1 l-asparaginases as pro-*S* and the absolute configuration of the transition state as *S*, in agreement with the (implicit) results of Lubkowski & Wlodawer (2019 ▸).

We note that the correlation diagram in the last panel of Fig. 3 ▸ shows, in addition to the well defined clusters for the Thr12 (green) and Thr89 (red) residues, several outliers belonging to the Thr12 group. Careful analysis of those cases reveals that they correspond to enzyme complexes crystallized with a substrate/ligand of the unnatural, opposite chirality (*i.e.*d-Asn/Asp), strongly indicating that d-asparagine is not a viable substrate of class 1 asparaginases.

### 3.3. Class 2 asparaginases

**Figure 5:**
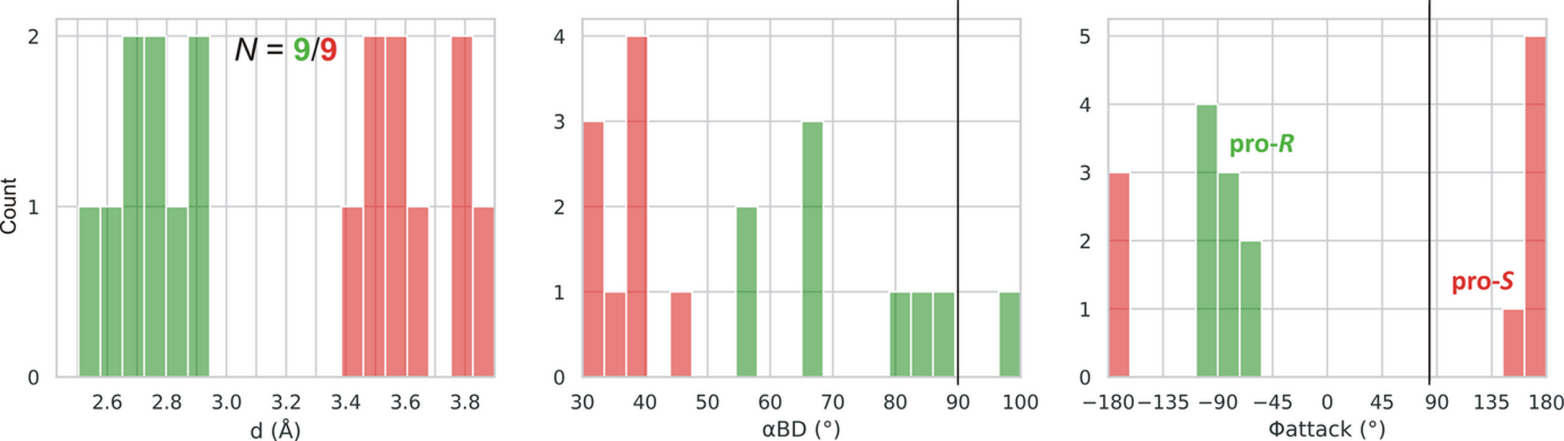
Histograms presenting the distribution of parameters *d*, α_BD_ and Φ_attack_ for the more (Thr179, green) and less (Thr230, red) likely nucleophile in class 2 l-asparaginases. Residue numbering of the reference protein EcAIII is used. The vertical black lines mark the expected values of α_BD_ and Φ_attack_.

The obvious nucleophile of Ntn-hydro­lases is the N-terminal residue of subunit β, Thr179 in the numbering of the *E. coli* protein EcAIII (Fig. 9 ▸), additionally confirmed by the PDB structures of the covalent β-acyl-enzyme ester intermediate (human enzyme 4o0h, Nomme *et al.*, 2014 ▸; EcAIII 8c0i, Janicki *et al.*, 2023 ▸). However, there are other Thr residues in the active site area, namely Thr197 (at the wrong end of the substrate), Thr230, and Thr232 (oriented away from the substrate, not shown in Fig. 9 ▸), in EcAIII numbering. In the standard interpretation of the reaction mechanism, the oxy­anion hole in EcAIII is formed by the N—H group of Gly231 and the O—H group of Thr230. It would be, therefore, not very likely that Thr230 could be the catalytic nucleophile as well, although its hydrogen bonding with the substrate Oδ1 atom could be viewed as substrate-assisted activation of the nucleophile. For the completeness of the analysis, we also included Thr230 in the calculations, assuming that the oxy­anion hole is then reduced to just the amide group of Gly231. The results of the analysis are presented in Supplementary Table S2 and as histograms in Fig. 5 ▸. The mean values of the geometric parameters are summarized in Table 1 ▸. The number of cases (9) is not very large, but the overall pattern is already clearly seen.

The first observation is that the Thr179 residue is much more likely as the catalytic nucleophile than Thr230. The foremost indication is from the *d* parameter, which for Thr179 is systematically shorter than for Thr230, covering a range of 2.52–3.89 Å. The second observation is that the angular parameters have wide distributions and are farther away from the expected values than in class 1. For example, the mean value of the α_BD_ angle for Thr179 is 74 (15)°, *i.e.* ∼16° from expectation.

The third observation is that the Φ_attack_ angle of Thr179 is negative, −83 (13)°, thus defining the chirality of the nucleophilic attack as pro-*R*, *i.e.* as occurring from the opposite side of the amide plane of the substrate relative to class 1 (and class 3, see below). This means that the direction of the nucleophilic attack during l-asparagine hydrolysis is not uniformly fixed in space, although it is constant in each class of asparaginases. We note that the absolute value of Φ_attack_ in this class is very close to the expectation of 84° given by Du *et al.* (2025 ▸).

### 3.4. Class 3 asparaginases

Class 3 asparaginases have two potential Ser nucleophiles in the active site, each part of an S-K tandem (Fig. 10 ▸). In the numbering of the reference ReAV enzyme from *R. etli*, they are Ser48 and Ser80. The more conspicuous candidate is Ser48, which in many apo structures has its OH group surrounded by three tetrahedrally arranged electron density peaks, provisionally interpreted as tightly hydrogen-bonded water molecules (Loch *et al.*, 2021 ▸). On the other hand, Ser80 has a very strained main-chain conformation, with the Ala79–Ser80 peptide bond far away from planarity (ω < 150°). The oxyanion hole of the ReAV enzyme is formed by the N—H amides of Ser48 and Ala266.

Close to the active-site S-K tandems, there is a Zn^2+^ binding site (substituted by Cd^2+^ in engineered variants of the ReAV enzyme). The metal cation is not involved in the catalytic mechanism (Pokrywka *et al.*, 2024 ▸) but instead serves as an anchor for the substrate, coordinating it via the α-carb­oxy and α-amino groups (Pokrywka *et al.*, 2025 ▸).

**Figure 6:**
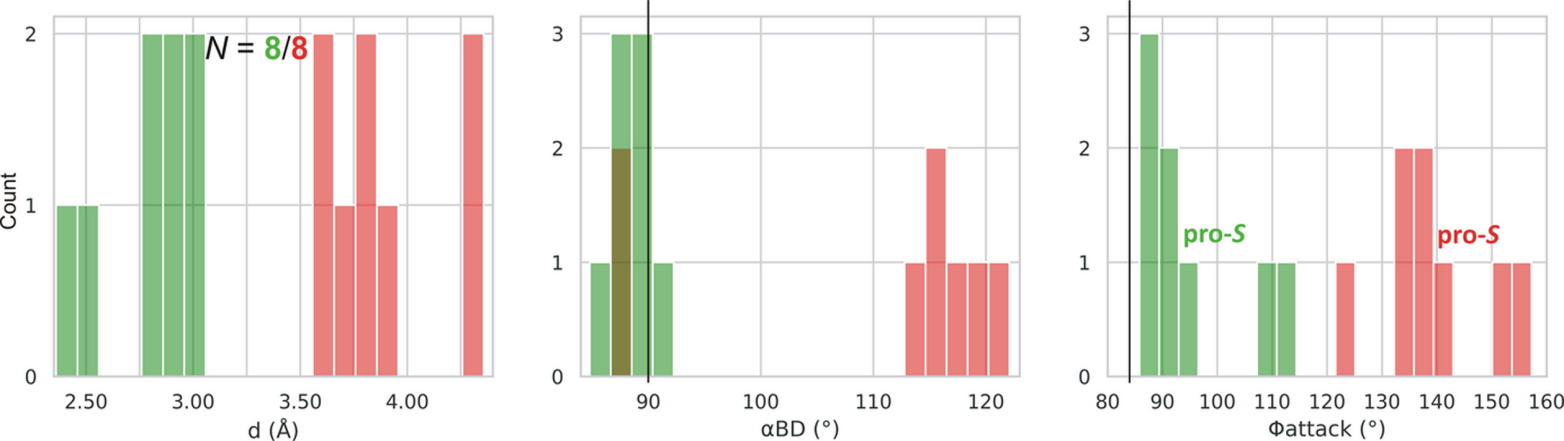
Histograms presenting the distribution of parameters *d*, α_BD_ and Φ_attack_ for the more (Ser48, green) and less (Ser80, red) likely nucleophile in class 3 l-asparaginases. Residue numbering of the reference protein ReAV is used. The vertical black lines mark the expected values of α_BD_ and Φ_attack_.

**Figure 7:**
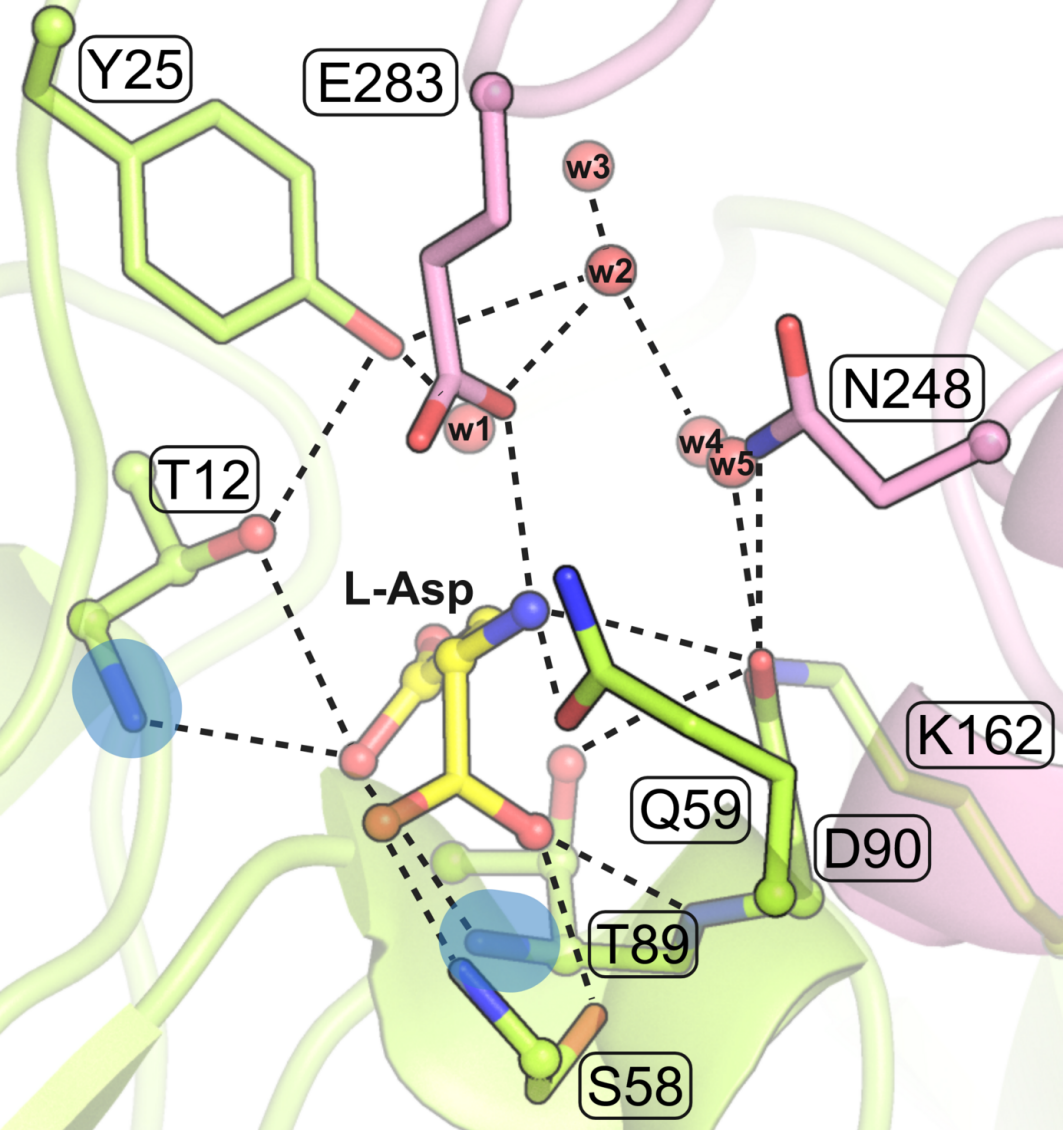
The active site of EcAII in complex with l-Asp from the PDB entry 3eca, chain A (Swain *et al.*, 1993 ▸). The two potential nucleophilic Thr residues, Thr12 and Thr89, are in ball-and-stick representation. Thr89 is part of the Thr89–Lys162–Asp90 T-K-D triad (labeled). The oxyanion hole is marked by blue ovals. Water molecules are shown as small red spheres. The most important hydrogen bonds are marked by black dashed lines. Note that in this type II enzyme, the hydrogen-bonded Thr12⋯Tyr25 residues come from the same subunit. In type I asparaginases the Tyr residue of this pair is contributed by the complementary subunit from the intimate dimer of the tetrameric assembly.

The recently published Bürgi–Dunitz analysis for three l-Asn complexes of ReAV and its mutants (Pokrywka *et al.*, 2025 ▸) is herein extended to include additional parameters and data (Table 1 ▸). The histograms of Fig. 6 ▸ provide unequivocal evidence in favor of Ser48 as the primary nucleophile, even though the number of cases (8) is rather limited. The two cases on the Φ_attack_ plot seen at ∼110°, separate from the cluster at ∼90°, can be identified as representing an l-Asn complex of ReAV without the metal cation, *i.e.* without the point of attachment for the substrate molecule. Consequently, the substrate molecules in this (dimeric) complex are more wobbly, and may be poorly presented to the catalytic apparatus. This is also reflected in the large spread of the Φ_attack_ distribution, 95 (10)°. The α_BD_ angle, on the other hand, has a sharp distribution near the expected value, 88 (2)°. The sign of the Φ_attack_ parameter defines the chirality of the nucleophilic attack as pro-*S*, as in class 1 asparaginases.

### 3.5. Short asparaginases (class 1 type s)

Currently, the PDB contains 10 structures of short-chain asparaginases, all of which come from *Rhodospirillum rubrum*, a bacterium with a versatile metabolism, including optional photosynthesis. Despite lacking the C-terminal oligomerization domain, they are homotetramers or dimers of intimate dimers. While at face value this quaternary state appears to be similar to the canonical oligomer architecture of class 1 asparaginases, in detail it is, however, very different. Four of these structures represent l-Asn/l-Asp complexes. The primary Thr16 nucleophile resides in one subunit and the catalytic Tyr21 relay is provided by the complementary subunit of the intimate dimer. This affiliation of the active-site Tyr residue suggests that short asparaginases should be grouped together with type I enzymes in class 1, even though the two intimate dimers in question are organized differently. The T-K-D (87-158-88) triad is also present, thus we were able to conduct our stereochemical analysis for those cases considering both Thr residues in the role of the nucleophile. The oxyanion hole is furnished by the N—H groups of the two Thr nucleophile candidates, as in all class 1 enzymes. The results are presented in Table S2, in graphical form in Fig. 4 ▸(*c*), and as a statistical summary in Table 1 ▸.

As in all class 1 cases, there is no ambiguity about the location of the primary nucleophile on the FGE loop. Considering the rather low number of type s examples (9), it is noted that they replicate the expected values of the α_BD_ and Φ_attack_ parameters very well, as 88 (5)° and 82 (4)°, respectively. The chirality of the nucleophilic attack in type s asparaginases is, of course, pro-*S*, as in all class 1 asparaginases.

The composition of the active site and the results of this SCM analysis allow us to classify short-chain l-asparaginases as a new type (s) in class 1. With such classification, we are able to expand the subset of class 1 cases from Section 3.2 and present a comprehensive statistical analysis as entry class 1 in Table 1 ▸. The α_BD_, α_LW_ and Φ_attack_ values of this all-inclusive set of class 1 l-asparaginases are in very good agreement with expectations at 90 (6), 1 (6) and 89 (6)°, respectively.

### 3.6. d-Asn as substrate for l-asparaginases

There are only a few l-asparaginase complexes with d-Asn/Asp ligands in the PDB, all belonging to class 1 type II. They could be easily intercepted in this analysis as outliers from the l-Asn/Asp clusters (Fig. 3 ▸). We can, therefore, conclude that d-Asn is not a substrate for l-asparaginases, at least for the enzymes with class 1 affiliation.

### 3.7. The case of l-glutaminases/l-asparaginases

The similar l-glutaminase activity (*i.e.* hydrolysis of the side-chain amide of l-glutamine) has been excluded experimentally for class 2 (Borek *et al.*, 2004 ▸) and class 3 (Sliwiak *et al.*, 2024 ▸) l-asparaginases. In class 1 enzymes, however, l-glutaminase activity has been always a serious consideration, especially for the type II antileukemic l-asparaginases, where l-glutaminase activity is generally regarded as an undesired source of adverse side effects^4^ (Ollenschläger *et al.*, 1988 ▸). In this perspective, l-glutaminase-free enzymes, *e.g.* WsA from *Wolinella succinogenes* or VcA from *Vibrio cholerae*, are considered preferable as antileukemic agents. On the other side of the spectrum, there are enzymes with dominating l-glutaminase activity, such as AGA from *Acinetobacter* or PGA from *Pseudomonas*. 

We analyzed separately all enzyme complexes (found only in class 1) crystallized with l-Gln or l-Glu in the active site. It is seen from Fig. 11 ▸ that most of those complexes are incompatible with the reaction stereochemistry, regardless of the primary nucleophile (Thr12 or Thr89 in EcAII numbering). An interesting explanation for the lack of l-glutaminase activity in (some of) those cases is illustrated in Fig. 12 ▸ by the PDB entry 5hw0 (beige), where the ErA Thr15 (corresponding to Thr12 in EcAII) nucleophile is swung away from the ligand and the l-Glu ligand is contorted out of the productive conformation illustrated by the l-Asp ligand of the same protein and the PDB entry 5f52.

In two instances, represented by the PDB entry 6wyy, the stereochemistry is actually quite plausible (α_BD_ ∼92°, Φ_attack_ ∼84°, *d* < 3 Å), and it is gratifying to note that this entry corresponds to the PGA enzyme from *Pseudomonas* 7A, with recognized l-glutaminase activity. As expected, the primary nucleophile in this case would be Thr20, corresponding to Thr12 of EcAII.

### 3.8. The geometry of the tetrahedral transition states

There are only two structures in the PDB, 6v2c and 6v2g, both representing class 1 type II, where the genuine tetrahedral transition state of the nucleophilic attack of l-asparaginase has been captured, in two protein chains in each crystal. Despite the fact that each of these transition states represents an alternative model (the other corresponding to the collapsed β-acyl ester intermediate), we decided to include them in our analysis as they have very good support from the corresponding electron density. In general, these examples fit very well the scenario of the nucleophilic attack painted in this work, as the terminal state on the reaction path. The only distortion is visible in the α_BD_ angle, which is deformed (or rather relaxed) from ∼90 to ∼110°.

### 3.9. Remarks on some structural errors and problems encountered in this analysis

While the main analytical part of this work was carried out automatically by a Python program developed for this purpose, the preparation of suitable input data required rather careful inspection of the individual PDB files. In the course of this inspection, a number of mistakes and errors (or special cases) were encountered, which are briefly summarized here. We note that the problems encountered in this work should be added to the list cataloged by Wlodawer *et al.* (2024 ▸). In most cases, trivial atom-naming inconsistencies, such as placing the Oδ2 atom of the l-Asp ligand in the oxyanion hole, could be easily corrected. There was also a case (6rue chain C; Timofeev *et al.*, 2020 ▸) where, despite evidence from the electron density map, the wrong rotamer of Thr14 (Thr12 equivalent) was modeled, with the methyl group poised for the nucleophilic attack. In some class 1 structures (*e.g.*6rue chain A), despite the presence of an l-Asp/l-Asn ligand and the primary nucleophile (Thr12 in EcAII numbering) in the active site, the active-site FGE loop was in a non-catalytic open conformation, and such cases had to be excluded from further analysis. However, the alternative Thr89 residue could still be included in the analysis.

### 3.10. On the architecture of the oxyanion hole

**Figure 8:**
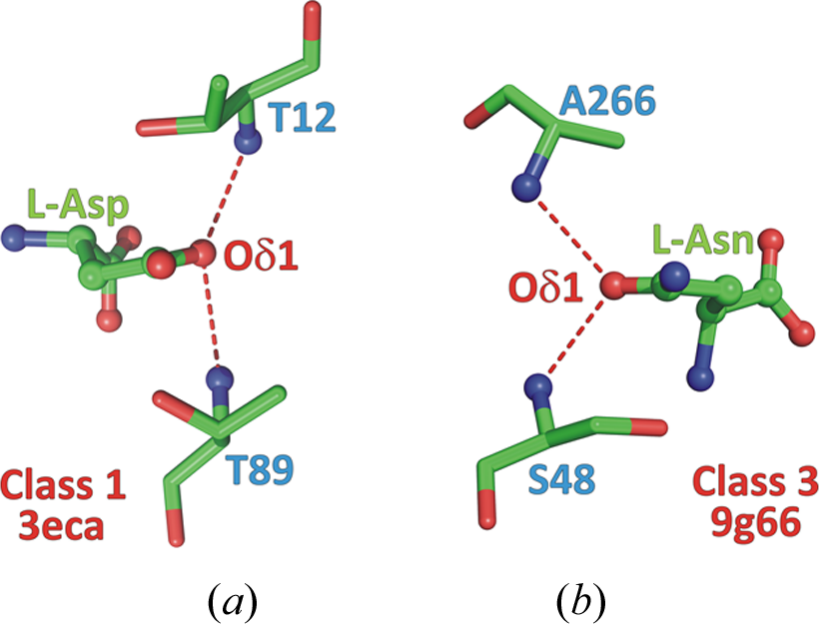
Arrangement of the N—H donors forming the oxyanion hole relative to the substrate amide (or product carb­oxy­lic in 3eca) group. The substrate/product amide/carb­oxy­lic plane is horizontal and roughly perpendicular to the plane of projection, with the C=Oδ1 bond pointing away from the viewer. The cases of (*a*) class 1 enzyme (PDB ID 3eca) and (*b*) class 3 enzyme (PDB ID 9g66).

**Figure 9:**
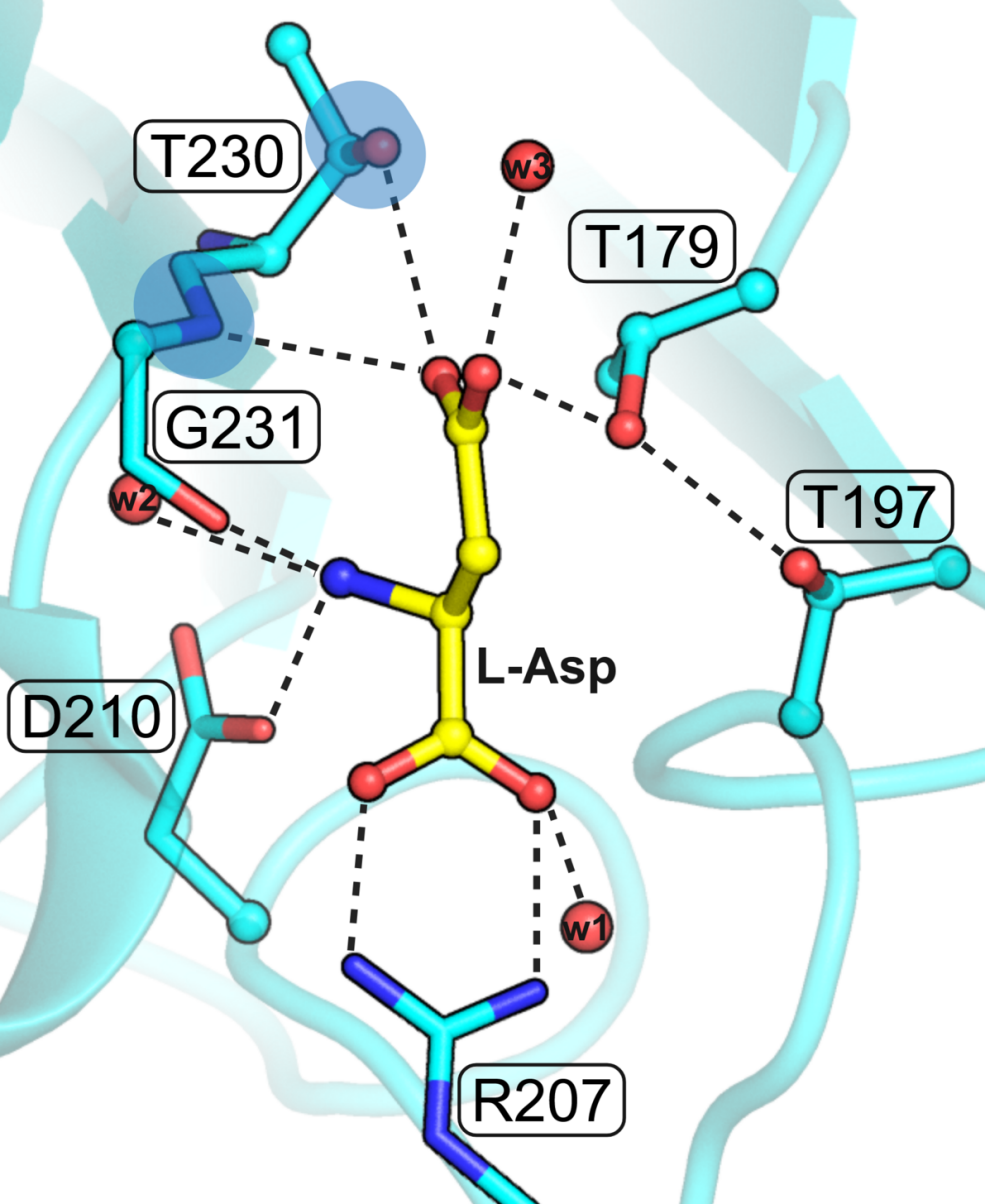
The active site of EcAIII in complex with l-Asp from the PDB entry 2zal, chain B (Michalska *et al.*, 2005 ▸). The N-terminal T179 nucleophile as well as two other Thr residues in the active site area (Thr197, Thr230) are in ball-and-stick representation. The oxyanion hole is marked by blue ovals. Water molecules are shown as small red spheres. The most important hydrogen bonds are marked by black dashed lines.

**Figure 10:**
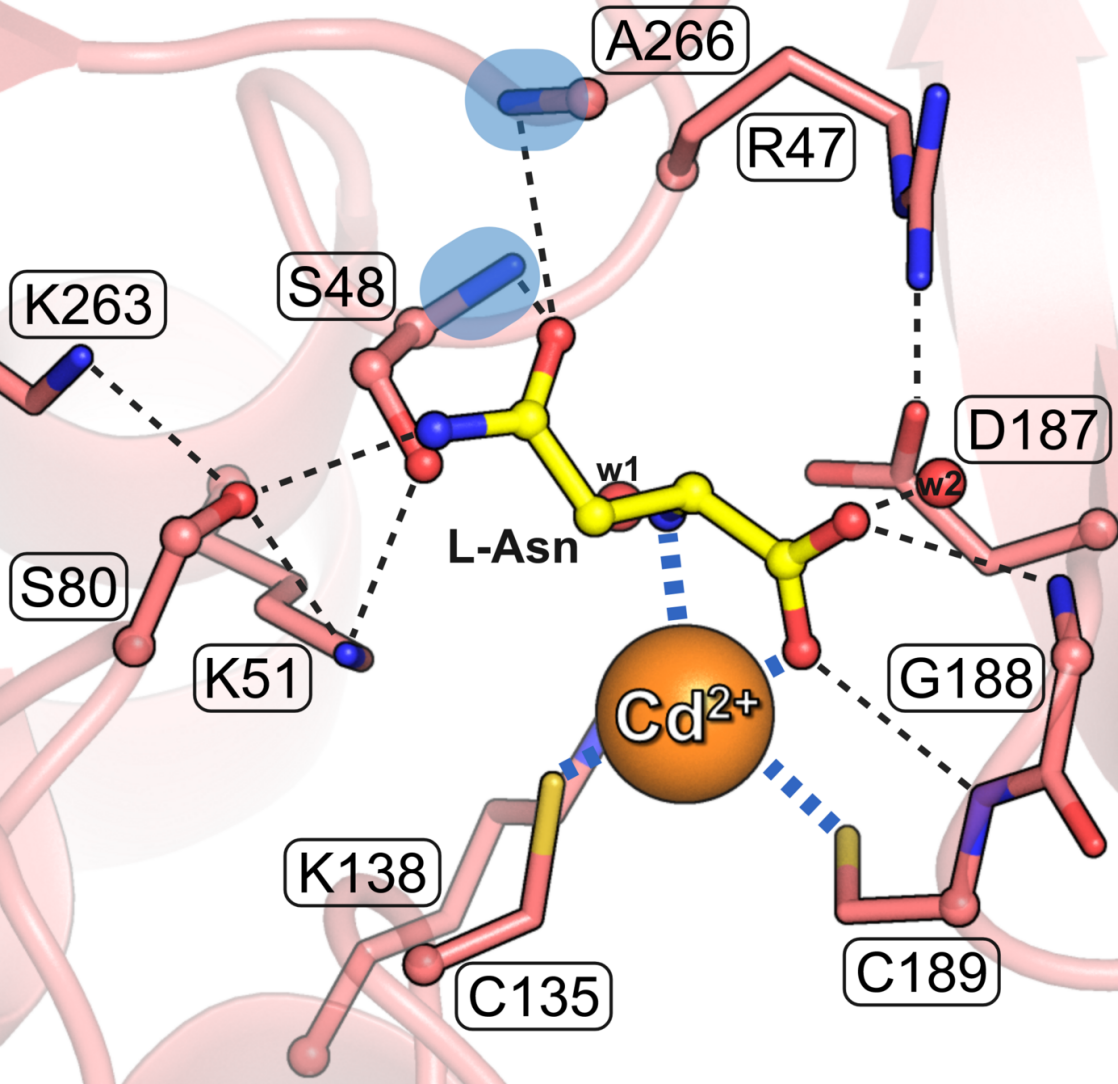
The active site of ReAV in complex with l-Asn from the PDB entry 9g66, chain A (Pokrywka *et al.*, 2025 ▸). The potential Ser48 and Ser80 nucleophiles from the two S–K tandems are in ball-and-stick representation. The oxyanion hole is marked by blue ovals. The Cd^2+^ cation (replacing Zn^2+^ in this variant) is marked by an orange sphere and water molecules are shown as small red balls. The most important hydrogen bonds are marked by black dashed lines and the coordination bonds around the cadmium cation are marked by blue dashes.

**Figure 11:**
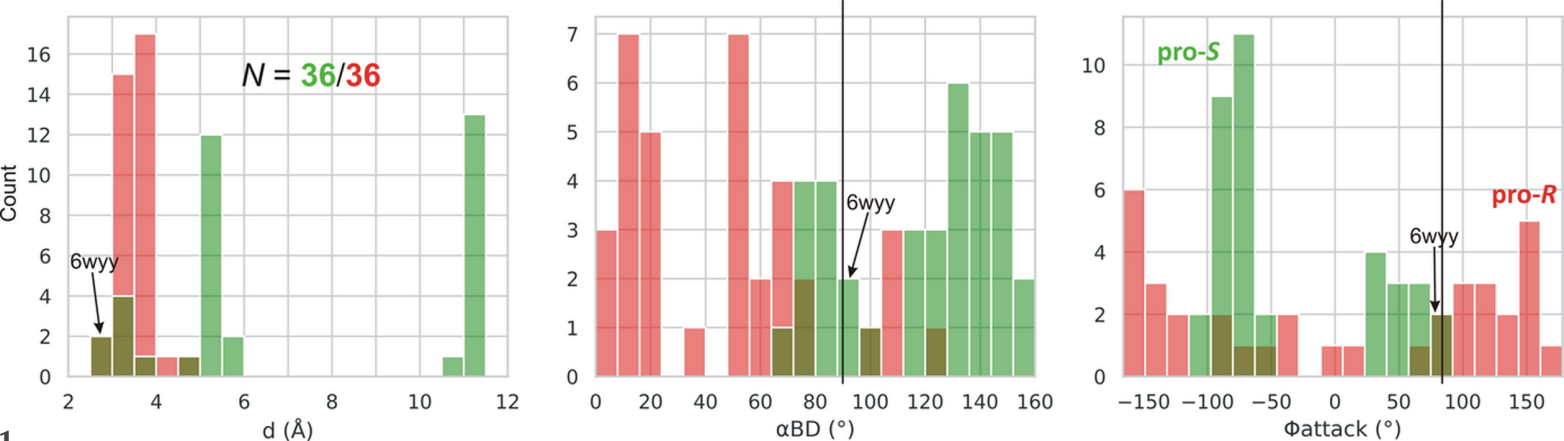
Histograms presenting the distribution of parameters *d*, α_BD_ and Φ_attack_ for l-Gln/Glu complexes of class 1 l-asparaginases. The highlighted PDB structure 6wyy corresponds to *Pseudomonas* 7A glutaminase-asparaginase PGA in complex with l-Glu.

**Figure 12:**
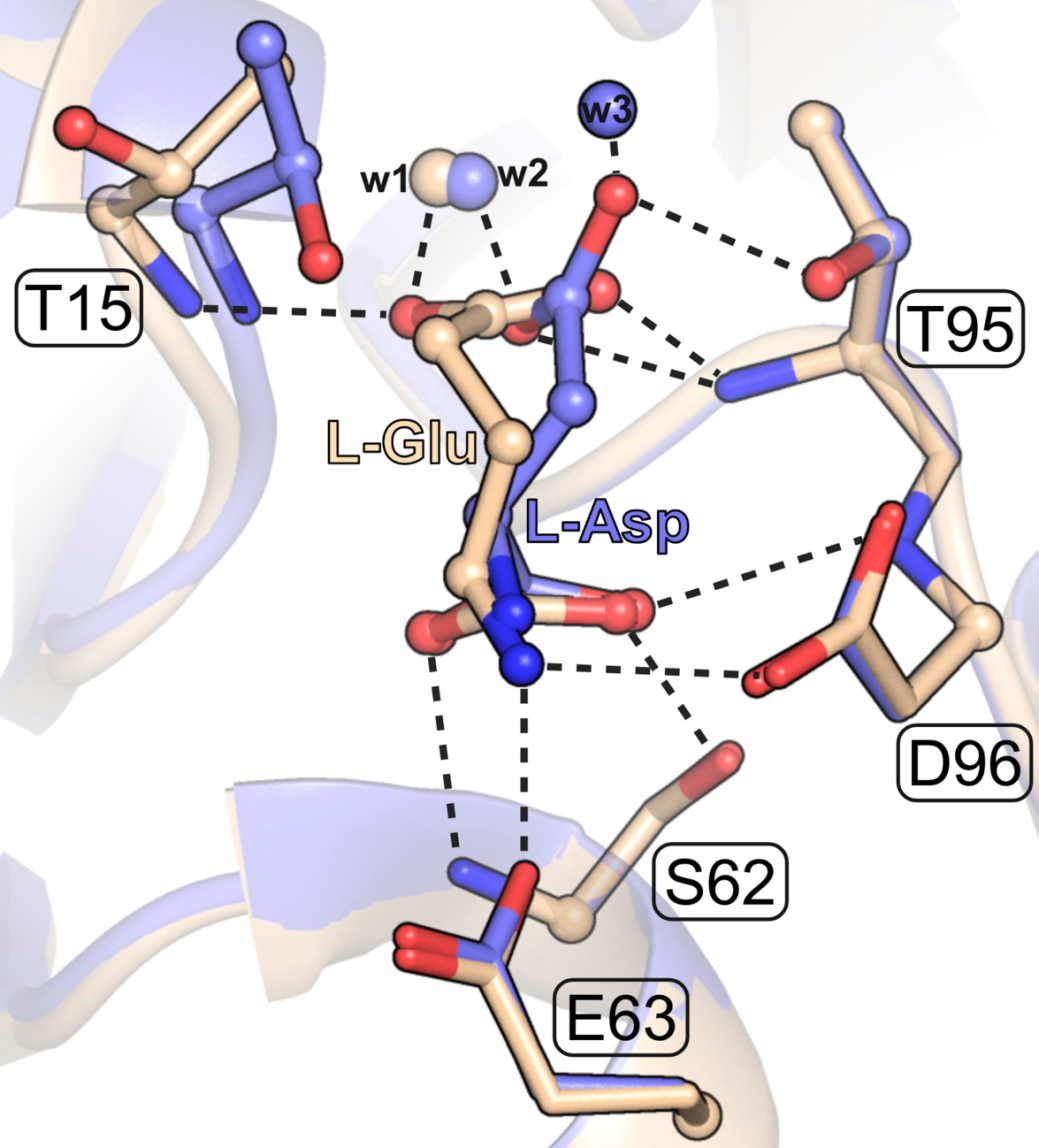
Superposition in the active site area of two ErA complexes, one with l-Asp (PDB ID 5f52, chain A, blue) and one with l-Glu (PDB ID 5wh0, chain C, beige). The ErA Thr15/Thr95 residues correspond to Thr12/Thr90 in EcAII. In the l-Glu complex, the functional group of the l-Glu ligand has a non-productive conformation and the Thr15 nucleophile is rotated away from the ligand.

The *sp*^2^ orbitals of the acceptor carbonyl O atom at the side-chain amide group of l-Asn are arranged trigonally in the plane of the amide group, at ∼120° to the C=O bond, and normally they would define the optimal directions of approach for suitable *X*—H hydrogen-bond donor groups. However, when we look at the directions of approach (to the amide plane) of the oxyanion hole components, especially in class 1 and class 3, where the donors are two main-chain N—H amides, we see a rather unfavorable constellation, with the N—H⋯O directions roughly perpendicular to the plane of the substrate amide group (Fig. 8 ▸). The situation becomes clear when we realize that the architecture of the oxyanion hole is optimized not for the inert substrate but for the transition state, when the carbonyl O atom assumes transiently a tetrahedral configuration, with the three ‘acceptor’ *sp*^3^ orbitals arranged spatially (and rotatable) around the C—O bond.

It is interesting to note that the oxyanion hole is often formed with the participation of the main-chain N—H amides donated by the nucleophilic groups in the active site (Fig. 8 ▸). In class 1, those amides come from the primary Thr12 nucleophile, as confirmed in this work, and from the second candidate, Thr89, which we could call an ‘optional’ or ‘reserve’ nucleophile. In class 3, one of the oxyanion hole amides is donated by the primary Ser48 nucleophile, as confirmed in this work.

### 3.11. Geometrical parameter distributions in the studied cases

Having a number of structural examples of the nucleophilic attack in l-asparaginases, we were able to calculate the mean values of the geometrical parameters used in this study and compare them among themselves and with similar distributions in other classes of nucleophilic attack on the carbonyl group. Naturally, the statistics are meaningful only for the —OH groups identified as the true primary nucleophiles of l-asparaginases (*i.e.* Thr12 in class 1, Thr179 in class 2 and Ser48 in class 3), although, for comparison, Table 1 ▸ lists the corresponding values for the alternative ‘nucleophiles’ as well. The results of the calculations are presented for the angles α_BD_, α_FL_, α_LW_ and the dihedral angle Φ_attack_. Obviously, no statistics are presented for the distance *d*, as it shrinks from very long to the limit of the covalent O—C bond as the nucleophilic reaction progresses. In class 1 the number of instances is very large and the selected parameters (except α_FL_) have approximately normal distributions. The statistics can, therefore, be calculated with confidence as the mean values and standard deviations of the distributions. In classes 2 and 3 the numbers of cases are rather small but still allow us to estimate approximate values of the statistical parameters.

The α_BD_ angle is close to its expected value of ∼90° in all classes and their subgroups, except in class 2, where it is by *ca* 16° smaller. Likewise the α_LW_ angle, expected to be ∼0°, shows the largest distortion (>10°) in class 2. This angle is, however, correlated with α_BD_ and is thus of limited value. As discussed before and attested by the large scatter of its values and huge standard deviations, the α_FL_ angle is not a useful parameter for the description of the nucleophilic attack. Finally, the Φ_attack_ angle is generally close to its expected value of ∼90° attributed to BDS, except in the group of short asparaginases (class 1 type s), where it is by ∼8° smaller and again in class 2, where it is by 7° off. These smaller values of Φ_attack_ are in better agreement with the value of 84° given by Du *et al.* (2025 ▸). The smaller Φ_attack_ captured for short asparaginases may indicate that indeed the catalytic mechanism in this group is a variant of that characteristic of class 1 in general.

The case of class 2 deserves a special mention as the Φ_attack_ angle has the opposite (*i.e.* negative) sign, defining the configuration of the transition state as *R*, *i.e.* opposite to the *S* configuration seen in all other cases. This means that in different classes of l-asparaginases the l-Asn substrate can be attacked from different ‘sides’ of the side-chain amide plane.

## 4. Conclusions

Our stereochemical analysis based on multiple crystal structures of enzyme–substrate/product complexes confirmed that the primary nucleophile in class 1 l-asparaginases is the Thr residue in the FGE loop. In accord with the Ntn affiliation, the primary nucleophile in class 2 is the N-terminal Thr of subunit β. In class 3, the primary nucleophile is the Ser residue from the ‘first’ (lower sequence position) S-K tandem. The emerging group of short asparaginases, despite the lack of the C-terminal domain, have structural, oligomeric and active-site features consistent with class 1 schemata, in which they have been included as type s. Accordingly, their primary nucleophile Thr residue is also part of the FGE loop, while the Tyr relay residue comes (as in type I enzymes) from the complementary subunit of the intimate dimer. The stereochemistry of the nucleophilic attack by short asparaginases may be, however, somewhat specific, as attested by the unusual value of the Φ_attack_ parameter. This supposition will have to be confirmed by further studies, as the number of cases of short asparaginase complexes is currently rather small (9) and limited to only one protein.

In classic application of the SCM principle to the nucleophilic attack on a carbonyl group, the stereochemistry of this attack is defined by the α_BD_ and α_FL_ angles. With the α_BD_ angle alone, the O_nuc_⋯C vector defines a cone in space around the C=O axis. In practical scenarios, when α_BD_ ≥ 90°, the cone is in fact centered around the extension of the O=C axis or is essentially flat (a circle perpendicular to the C=O bond). To select the concrete O_nuc_ point on the α_BD_ cone, the α_FL_ angle was introduced, in theory fixing the azimuth of the O_nuc_ nucleophile, *i.e.* the angle between the C–P line through its projection on the electrophile plane and the extension of the O=C line. The trouble with the α_FL_ definition is that when the O_nuc_⋯C vector is nearly perpendicular to the electrophile plane, α_FL_ cannot be calculated because the C–P vector becomes 0. Lubkowski & Wlodawer (2019 ▸) provided a remedy, without giving a reason explicitly, by introducing the α_LW_ (this label was coined in the work of Pokrywka *et al.*, 2025 ▸) angle between the C⋯O_nuc_ vector and its projection on a plane perpendicular to the electrophile plane and passing through the C=O bond (plane Ω). This angle also fixes the position of the nucleophile on the α_BD_ cone without the shortcoming of the α_FL_ definition. However, such a definition of α_LW_ becomes mathematically deficient as the C⋯O_nuc_ vector rotates towards the electrophile plane, in which case α_LW_ becomes 100% correlated with the α_BD_ angle (sin α_LW_ = sin α_BD_ · cos τ, where τ is the dihedral angle between the electrophile plane and the O_nuc_⋯C=O plane). Under normal circumstances, however, when the deviation of the C⋯O_nuc_ vector from the Ω plane is small, α_LW_ is a useful parameter. Nevertheless, in this work we propose to characterize the nucleophilic attack in l-asparaginases by the attack distance *d*, the two angles α_BD_ and Φ_attack_, and a letter (*S*/*R*) designating the chirality of the transition state. The latter designation expresses the sign of the Φ_attack_ angle (Φ_attack_ > 0°: *S*; Φ_attack_ < 0°: *R*) but we have decided to keep it despite its redundancy. The Φ_attack_ torsion angle O_nuc_–O=C_el_—C has been borrowed from Herschlag *et al.* (Du *et al.*, 2025 ▸), with some modification.

During macromolecular crystal structure refinement, especially at lower resolution, the structural parameters encoded in the diffraction data are sometimes ‘fighting’ against the idealized values enforced by different types of stereochemical restraints. This aspect is also visible in our analysis. The pyramidalization parameter Δ is practically meaningless because molecular planarity (such as that of the amide group) is usually tightly restrained. This is why we see Δ ≃ 0 Å also in the cases when the nucleophile approach O_nuc_⋯C_el_ is very short (*e.g.d* = 2.36 Å, Δ = −0.05 Å). However, when *d* gets extremely short, C_el_ pyramidalization is clearly visible despite the restraining counter effect (*e.g.d* = 2.25 Å, Δ = 0.15 Å). van der Waals repulsions are less tightly restrained and, therefore, *d* is a much more sensitive and reliable indicator of the progress on the reaction pathway. Intermolecular angles and torsion angles are not (explicitly) restrained and, thus, the angular parameters α_BD_ and Φ_attack_ are good descriptors for SCM analysis. The stereochemical restraints have less influence at high resolution. It is, therefore, important to ensure that crystal structures are determined (and deposited in the PDB) at the highest possible resolution.

## Supplementary Material

Supplementary Table S1. DOI: 10.1107/S2052252525010826/min5003sup1.xlsx

Supplementary Table S2. DOI: 10.1107/S2052252525010826/min5003sup2.xlsx

## Figures and Tables

**Table 1:** Geometric parameter statistics (mean μ with standard deviation of the distribution σ given in parentheses) in different groups of L-asparaginases *N* is the number of cases.

Enzyme group	*N*	α_BD_ (°)	α_FL_ (°)	α_LW_ (°)	Φ_attack_ (°)
Class 1 type I	14	87.0 (6.7)	48.6 (127.1)	−2.3 (3.9)	92.3 (4.0)
Class 1 type II	128	90.1 (6.1)	−25.4 (101.0)	0.8 (6.3)	89.2 (6.3)
Class 1 type s	9	87.7 (4.9)	−107.1 (36.3)	7.7 (4.2)	82.4 (4.2)
Class 1 type I + II	142	89.8 (6.2)	−18.1 (105.7)	0.5 (6.1)	89.5 (6.2)
Class 1	151	89.6 (6.1)	−23.4 (104.9)	0.9 (6.3)	89.1 (6.3)
Class 2	9	73.7 (15.0)	−28.6 (148.5)	10.5 (7.2)	−83.5 (13.4)
Class 3	8	88.2 (2.0)	33.6 (117.4)	7.3 (8.5)	95.1 (10.2)
All	168	88.7 (7.6)	−21.0 (108.1)	1.7 (6.8)	87.0 (15.2)†

† Arithmetic mean taken over absolute values of all instances.

## Data Availability

The Python program *BD* used for the SCM analysis is available from MG on request. Supplementary Table S1: PDB codes (pdb_id) of the entries used in this analysis, with basic bibliographic and quality information, including resolution (Å), extracted from the PDB headers, *R*_free_, clashscore, and Ramachandran, side chain and RSRZ outliers (%), extracted, if available, from the PDB validation reports, arranged according to classes, types and characteristic ligands. Supplementary Table S2: Excel file with geometrical parameters calculated for PDB entries, identified by their ID codes (pdb_id), corresponding to substrate/product complexes of l-asparaginases used in this analysis, arranged according to classes, types and characteristic ligands. ‘Primary’ refers to the main nucleophile identified in this work, Thr12 in class 1, Thr179 in class 2 and Ser48 in class 3, in residue numbering of EcAII, EcAIII and ReAV, respectively. ‘Secondary’ refers to the alternative nucleophile, Thr89 in class 1, Thr230 in class 2 and Ser80 in class 3, in the same numbering reference. The geometrical parameters *d*, α_BD_, *d*P, α_FL_, α_LW_, Φ_attack_ and Δ are described in Section 2.4. *R*/*S* defines the chirality of the nucleophilic attack.

## Footnotes

1 Du *et al.* (2025 ▸) also cite a value of ∼90° (BDS), estimated for Φ_attack_ from the work of Bürgi *et al.* (1974 ▸).

2 The 9sdk structure will be published elsewhere. Its basic statistical parameters are included in Table S1.

3 When we view the tetrahedral transition state along the Cγ–Cβ vector, then the Oδ1–Oγ–Nδ2 sequence is read in the counterclockwise direction (Fig. 2 ▸).

4 It must be noted, however, that another school of thought considers l-glutaminase activity a welcome feature of antileukemic l-asparaginases, as it would also deplete the level of free l-Gln, which in certain biochemical reactions may substitute for l-Asn (Holcenberg *et al.*, 1975 ▸; Spiers & Wade, 1976 ▸).
